# The Effectiveness of Adolescent-Focused Therapy and Family-Based Therapy for Anorexia Nervosa

**DOI:** 10.1177/00332941241226687

**Published:** 2024-01-18

**Authors:** Marni P. Stewart, Oliver Baumann

**Affiliations:** School of Psychology, 3555 Bond University, Gold Coast, QLD, Australia

**Keywords:** Eating disorder, addiction, disability & trauma, mental health, mental & physical health, psychotherapy, mental health, mental & physical health, self-esteem, self-worth, social perceptions, mental & physical health, risk perception, social perceptions, mental & physical health, anorexia nervosa, adolescent, young adulthood, family-based therapy, adolescent-focused therapy

## Abstract

Anorexia Nervosa is the most deadly mental illness due to the high mortality and relapse rates after reaching remission. The systematic review investigated the effectiveness of two empirically validated interventions (Family-Based Therapy [FBT] and Adolescent-Focused Therapy [AFT]) for an adolescent or young adult living with Anorexia Nervosa to reach partial or full remission and expected weight ratios. Twelve studies published between 1994 and 2015 were evaluated and indicated that FBT resulted in significant weight gain and higher partial and full remission rates than AFT, demonstrating its superiority in treating AN in adolescents and young adult samples, in one instance, at least up to 4 years. Despite FBT and AFT delivery, a significant proportion of participants did not achieve their target weight or full remission, indicating that both treatments may not be effective in all circumstances.

Across the globe, approximately 70 million people live with an eating disorder (McGorry, 2020). *Eating disorders* are serious, complex, and potentially life-threatening mental illnesses (McGorry, 2020). *Anorexia Nervosa* (AN) is a specific eating disorder categorised by extremely low body weight and the distorted view of the patients’ body image (American Psychiatric Association, 2013). Cognitions revolve around an excessive fear of weight gain or persistent lack of recognition of the seriousness of low body weight, evidenced by extreme dieting behaviours, which often co-occur with intense elevated exercise (American Psychiatric Association, 2013). There are two subtypes of AN: Restrictive and Binge-Eating/Purging. Rigid rules (e.g., only eating food of a specific colour) often accompany extreme dieting and undue amounts of exercise, characterising Restrictive AN (American Psychiatric Association, 2013). Whereas, Binge-Eating/Purging AN sees a person also engage in periods of over-eating, self-prompted vomiting, and abusing laxatives, diuretics, or enemas (American Psychiatric Association, 2013).

Anorexia nervosa is the most deadly mental illness as 25% of individuals that reach remission are likely to relapse, and the mortality rate is ten times higher for young people between 15 and 24 years than their same-aged peers (Arcelus et al., 2011; Keski-Rahkonen et al., 2008). Anorexia Nervosa is prevalent in up to 3.0% of adolescent females, with a lifetime prevalence of 0.3% (Arcelus et al., 2011). Male prevalence is less understood; however, clinical populations typically indicate a ratio of 10:1 female-to-male, with a lifetime prevalence up to 0.5% (American Psychiatric Association, 2013; Arcelus et al., 2011). Nevertheless, AN commonly goes undiagnosed, with only 25% of individuals experiencing body image and dieting concerns obtaining professional support (Arcelus et al., 2011).

## Anorexia Nervosa Treatments

Family-Based Therapy (FBT) is an evidence-based psychotherapy incorporating family members as the focus of the treatment process. It is commonly used to treat eating disorders, centring on weight restoration (Chen et al., 2016). The FBT used today draws on Behavioural Family Systems Therapy developed by Robin and Foster (1989), where clients are seen as incompetent in meeting their biological needs. The approach encourages the client to see their strengths and individuality, simultaneously empowering parents to take control of the child’s nutrition and daily food intake to promote weight gain (Chen et al., 2016). Although FBT is the recommended first-line approach to treating AN in adolescents and young adults, concerns have been raised regarding its efficacy and replicability when treating individuals across the lifespan (Chen et al., 2016).

Alternatively, Adolescent-Focused Therapy (AFT) is an individual psychotherapy commonly used to treat eating disorders in teenagers and young adults (Lock et al., 2010). The AFT used today draws on Ego-Orientated Individual Therapy developed by Garner and Garfinkel (1984), as the client demonstrates a reduced self-concept, characterising self-control as a biological need. It explores the client’s strengths and abilities, challenging cognitive distortions concerning body image and dietary restraint in a supportive context (Lock et al., 2010). However, there is limited research exploring the efficacy of AFT and related factors influencing the likelihood of remission.

There is a plethora of research and well-established relationships concerning FBT for AN treatment and remission rates in adolescents and young adults, yet, there is limited research exploring the efficacy of AFT for the likelihood of remission. Moreover, no research has systematically examined the difference between FBT and AFT in remission rates and treatment outcomes. Therefore, the present systematic review aims to compare the efficacy of AFT and FBT in treating adolescents and young adults diagnosed with AN to reach and maintain full remission and inform psychotherapists working in the field of associated considerations. The research question for the study is: Is there a difference in the effectiveness of FBT and AFT for an adolescent or young adult living with Anorexia Nervosa to reach partial or full remission and expected weight ratios?

## Method

### Search Strategy

The method of the present review aligns with the statement of Preferred Reporting Items for Systematic Reviews and Meta-Analyses (PRISMA; Moher et al., 2009). A two-person selection approach was applied. An empirical literature search was undertaken using the databases PubMed and PsychINFO on June 15, 2022 and October 10, 2022, using the following terms: *Adolescent Focused Therapy* OR *AFT* AND *Individual Therapy* OR *Individualised Focused Therapy* AND *Family Based Therapy* OR *FBT* AND *Anorexia Nervosa* (please see Table 1).

**Table 1.** Details of Database Access and Coverage.

Database	Type	Coverage Period	Number of Records Retrieved	Access Restriction
PubMed	Biomedical	Inception – June 14, 2022	44	None
PsycINFO	Psychology	Inception – June 14, 2022	471	Subscription needed

### Eligibility Criteria

Several preconditions were determined for inclusion of a study in the present research: (1) Focus on AN as the sole eating disorder; (2) A diagnosis of AN to use psychometrically sound anthropometric or self-report measure, or interview protocols using the DSM-III-R, DSM-IV, or DSM-V, with or without the amenorrhea criteria (American Psychiatric Association, 1987; 1994; 2013); (3) Comparison of AFT and FBT in an outpatient program; (4) Participants were adolescent or young adults (11 years to 25 years old), of any gender; (5) Any date; (6) Any research design. Review articles and book chapters were excluded from the present study to explore the practical potentials and limitations of AFT and FBT on remission rates rather than exploring the theoretical constructs.

## Results

### Study Selection

**Figure 1.**
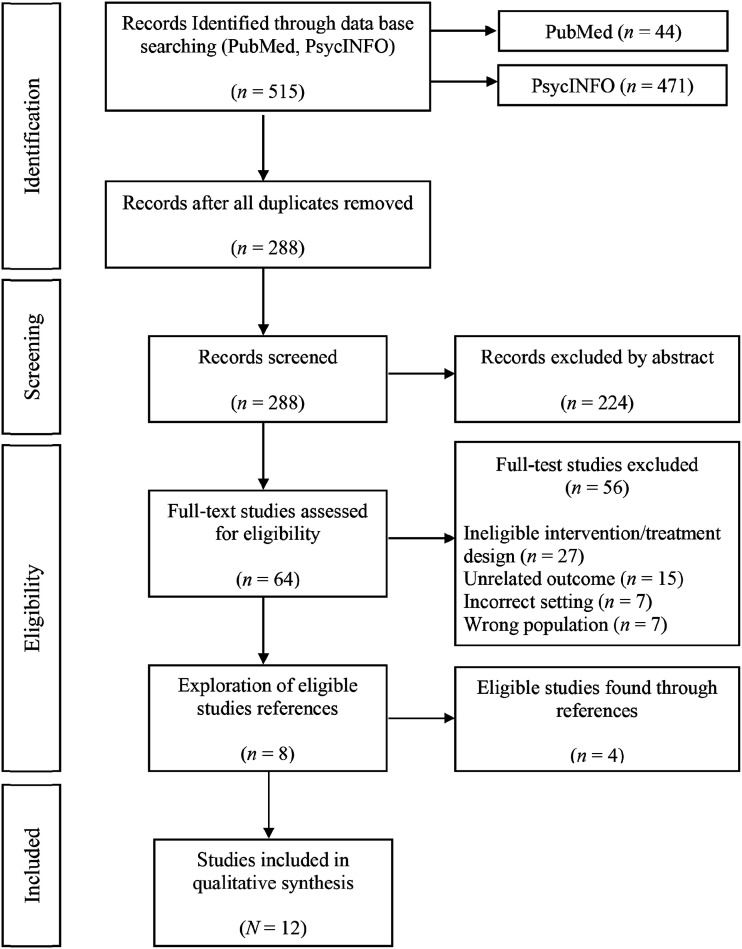
PRISMA flowchart illustrating article selection.

The initial search identified 515 articles, with 288 remaining after duplicate removal. (See Figure 1). Abstract screening identified 224 articles that did not compare AFT and FBT or were either a review or book chapter. Remaining studies (*n* = 64) were screened by full text. Studies were removed if they did not use AFT or FBT treatments (*n* = 27), did not measure remission rates (*n* = 15), were not an outpatient program (*n* = 7), or participants were not within the required age range (*n* = 7). Of the remaining eight studies, reference lists indicated four further studies which met inclusion criteria, and these were retrieved and included in the final sample of studies (*N* = 12).

### Study Characteristics

#### Participants

Studies in the final sample were published between 1994 and 2015. Sample sizes ranged from 22 to 121 participants, between the ages of aged 11 and 20 years old. Predominantly, participants were females (91%), followed by males (9%). All studies used randomised control trials with pre and post-testing to assess remission and associated factors.

#### Diagnosis

Diagnosis of AN in the studies used the DSM-III-R (*n* = 3) or DSM-IV (*n* = 9). All studies measured Body Mass Index (BMI). Also, nine studies employed the percentage of expected body weight (EBW), using the Centres for Disease Control and Prevention norms for age and gender and the Eating Disorder Examination (EDE, v12; Cooper et al., 1989; Schantz & Tsang, 2003). The remaining three studies administered the Eating Attitude Test, version 26 (EAT-26; Garner & Garfinkel, 1979), Body Shape Questionnaire (BSQ; Cooper et al., 1987), and three scales of the Eating Disorder Inventory (Garner et al., 1983).

### Scales

Across the studies, psychometrically sound measures of co-morbidities were used (see Table 2), including: Beck Depression Inventory (BDI; Beck et al., 1988); Rosenberg Self-Esteem Scale (RSES; Rosenberg, 1965); Schedule for Affective Disorders and Schizophrenia for School-Age Children (K-SADS; Kaufman et al., 1997 ); Yale-Brown-Cornell Eating Disorder Scale (YBC-EDS; Mazure et al., 1994); Parent-Adolescent Relationship Questionnaire (PARQ; Robin et al., 1990); General Self-Efficacy Scale (GES; Sukmak et al., 2001); Working Alliance Inventory (WAI; Tichenor & Hill, 1989); Work and Social Adjustment Scale (WSAS; Mundt et al., 2002); and Family Assessment Device (FAD; Epstein et al., 1983).

**Table 2.** Overview of the Selected Studies, Participants, Treatment, Methodology, Key Results, and Key Limitations.

Study	Diagnostic Manual and Diagnosis	AFT Participant Description	FBT Participant Description	Measures	Design	Points of Assessment	Key Results	Key Limitations
Accurso et al. (2014) Is weight gain really a catalyst for broader recovery? The impact of weight gain on psychological symptoms in the treatment of adolescent anorexia nervosa	DSM-IV excluding the amenorrhea criterion Anorexia Nervosa Subtype Binge-Eating/Purging *n* = 21 Restrictive *n* = 100	*N* = 60 Gender Female *n* = 56 Male *n* = 4 Age 12 to 18 years *M* = 14.7 Duration of illness 10.3 months Previous hospitalisations 29 days Using psychotropic medications *n* = 11 BMI percentile *M* = 5.2 Family status Intact *n* = 46 Separated *n* = 14	*N* = 61 Gender Female *n* = 54 Male *n* = 7 Age 12 to 18 years *M* = 14.1 Duration of illness 12.3 months Previous hospitalisations 25 days Using psychotropic medications *n* = 9 BMI percentile *M* = 7.2 Family status Intact *n* = 49 Separated *n* = 12	Expected Body Weight (EBW) Body Mass Index (BMI) The Eating Disorder Examination (EDE, v12; Cooper et al., 1989) The Beck Depression Inventory (BDI; Beck et al., 1988) The Rosenberg Self-Esteem Scale (RSES; Rosenberg, 1965)	Multisite Randomised Control Trial (RCT)	Pre treatment End of treatment 6 months post treatment 12 months post treatment	Eating disorder symptoms significantly improved for individuals in both AFT and FBT from pre-treatment to 12 month follow up. Depressive and dietary restraint symptoms reduced the most, while worries concerning body shape and weight improved least. Furthermore, self-esteem did not progress.	20 participants were taking psychotropic medications at baseline. 29 participants met criteria for a current co-morbid psychiatric disorder.
Bryne et al. (2015) An exploratory examination of patient and parentalself-effiicacy as predictors of weight gain in adolescents with anorexia nervosa	DSM-IV excluding the amenorrhea criterion Anorexia Nervosa	*N* = 60 Gender Female *n* = 56 Male *n* = 4 Age 12 to 18 years *M* = 14.7 Duration of illness 10.3 months Previous hospitalisations 29 days Using psychotropic medications *n* = 11 BMI percentile *M* = 5.2 Family status Intact *n* = 46 Separated *n* = 14	*N* = 61 Gender Female *n* = 54 Male *n* = 7 Age 12 to 18 years *M* = 14.1 Duration of illness 12.3 months Previous hospitalisations 25 days Using psychotropic medications *n* = 9 BMI percentile *M* = 7.2 Family status Intact *n* = 49 Separated *n* = 12	Expected Body Weight (EBW) Body Mass Index (BMI) The Eating Disorder Examination (EDE, v12; Cooper et al., 1989) The Beck Depression Inventory (BDI; Beck et al., 1988) The Rosenberg Self-Esteem Scale (RSES; Rosenberg, 1965) Family status (i.e., separated/divorced parents) Schedule for Affective Disorder and Schizophrenia for School-Age Children; K-SADS; (Kaufman et al., 1997)	Multisite Randomised Control Trial (RCT)	Pre treatment End of treatment 6 months post treatment 12 months post treatment	Families receiving FBT indicated more notable increases in parental self-efficacy, forecasting subsequent weight gain. However, participants’ self-efficacy ratings did not predict their weight gain.	Mother and father self-efficacy scores were combined to create ‘Parental self-efficacy’. The use of weight gain as the outcome measure, rather than using the remission criteria identified (e.g., full remission <95 EBW and partial remission <85 EBW), as weight gain is not equivalent with recovery
Ciao et al., 2015 Family functioning in two treatments for adolescent anorexia nervosa	DSM-IV excluding the amenorrhea criterion Anorexia Nervosa Subtype Binge-Eating/Purging *n* = 21 Restrictive *n* = 100	*N* = 60 Gender Female *n* = 56 Male *n* = 4 Age 12 to 18 years *M* = 14.7 Duration of illness 10.3 months Previous hospitalisations 29 days Using psychotropic medications *n* = 11 BMI percentile *M* = 5.2 Family Status Intact *n* = 46 Separated *n* = 14	*N* = 61 Gender Female *n* = 54 Male *n* = 7 Age 12–18 years *M* = 14.1 Duration of illness 12.3 months Previous hospitalisations 25 days Using psychotropic medications *n* = 9 BMI percentile *M* = 7.2 Family status Intact *n* = 49 Separated *n* = 12	Expected Body Weight (EBW) Body Mass Index (BMI) The Eating Disorder Examination (EDE, v12; Cooper et al., 1989) The Beck Depression Inventory (BDI; Beck et al., 1988) The Rosenberg Self-Esteem Scale (RSES; Rosenberg, 1965) Yale-Brown-Cornell Eating Disorder Scale (YBC-EDS; Mazure et al., 1994) General Self-Efficacy Scale (GES; Sukmak et al., 2001) Work and Social Adjustment Scale (WSAS; Mundt et al., 2002) Family Assessment Device (FAD; Epstein et al., 1983)	Multisite Randomised Control Trial (RCT)	Pre treatment End of treatment	Developments in a variety of aspects relating to family functioning were uniquely associated with full remission at the end of treatment, irrespective of the initial level of family functioning in both AFT and FBT treatments; although FBT demonstrated more positive effects when compared to AFT	Does not explore family functioning’s relationship to remission rates after the end of treatment (i.e., no reference to 6 month or 12 month follow up data)
(Forsberg, 2011) The relationship between therapeutic alliance and treatment outcome in a comparative study of individuality and family therapy for adolescents anorexia nervosa	DSM-IV excluding the amenorrhea criterion Anorexia Nervosa	*N* = 40 Gender NA Age 12 to 18 years	*N* = 38 Gender NA Age 12 to 18 years	Expected Body Weight (EBW) Body Mass Index (BMI) The Eating Disorder Examination (EDE, v12; Cooper et al., 1989) Working Alliance Inventory (WAI; Tichenor & Hill, 1989)	Multisite Randomised Control Trial (RCT)	Pre treatment End of treatment 6 months post treatment 12 months post treatment	The therapeutic relationship was stronger in AFT than FBT. Additionally, a strong alliance may help individuals achieve partial remission by end of treatment, yet did not predict full remission.	The study only included 78 out of the 121 participants from Lock et al. (2010) primary data collection, due to audio quality difficulties. It was highlighted that the smaller number of participants was of concern as the study was not powered adequately to address their research question.
Forsberg et al., 2014 Therapeutic alliance in two treatments for adolescent anorexia nervosa	DSM-IV excluding the amenorrhea criterion Anorexia Nervosa	*N* = 40 Gender NA Age 12 to 18 years	*N* = 38 Gender NA Age 12 to 18 years	Expected Body Weight (EBW) Body Mass Index (BMI) The Eating Disorder Examination (EDE, v12; Cooper et al., 1989) Working Alliance Inventory (WAI; Tichenor & Hill, 1989)	Multisite Randomised Control Trial (RCT)	Pre treatment End of treatment 6 months post treatment 12 months post treatment	Therapeutic relationship is achievable across AFT and FBT; however, rapport was stronger in AFT than FBT, yet did not predict full remission.	The study highlighted that it was not able to assess the relationship between rapport and symptom change before sessions 3 and 4, or after session 5, significantly reducing the amount if data available.
Le Grange et al., 2012 Moderators and mediators of remission in family-based treatment and adolescent focused therapy for anorexia nervosa	DSM-IV excluding the amenorrhea criterion Anorexia Nervosa	*N* = 60 Gender Female *N* = 56 Male *N* = 4 Age 12 to 18 years *M* =14.7 Duration of illness 10.3 months Previous hospitalisations 29 days Using psychotropic medications *n* = 11 BMI percentile *M* = 5.2 Family status Intact *n* = 46 Separated *n* = 14	*N* = 61 Gender Female *n* = 54 Male *n* = 7 Age 12 to 18 years *M* = 14.1 Duration of illness 12.3 months Previous hospitalisations 25 days Using psychotropic medications *n* = 9 BMI percentile *M* = 7.2 Family status Intact *n* = 49 Separated *n* = 12	Expected Body Weight (EBW) Body Mass Index (BMI) The Eating Disorder Examination (EDE, v12; Cooper et al., 1989) The Beck Depression Inventory (BDI; Beck et al., 1988) The Rosenberg Self-Esteem Scale (RSES; Rosenberg, 1965) Family status (i.e., separated/divorced parents), Schedule for Affective Disorder and schizophrenia for school-Age Children; K-SADS; (Kaufman et al., 1997)	Multisite Randomised Control Trial (RCT)	Pre treatment End of treatment 6 months post treatment 12 months post treatment	Participants with higher unrealistic perceptions and psychological symptoms responded better to FBT than AFT. Also Adolescents with Restrictive AN responded better to treatment overall	Participants were treatment seeking, medically stable, and had low levels of comorbidity with other psychological disorders. Such influences may restrict the generalisability of findings
Le Grange, Accurso et al. (2014) Early weight gain predicts outcome in two treatments for adolescent anorexia nervosa	DSM-IV excluding the amenorrhea criterion Anorexia Nervosa AN Subtypes Binge-Eating/Purging *n* = 21 Restrictive *n* = 100	*N* = 60 Gender Female *n* = 56 Male *n* = 4 Age 12 to 18 years *M* = 14.7 Duration of illness 10.3 months Previous hospitalisations *M* = 29 days Using psychotropic medications *n* = 11 BMI percentile *M* = 5.2 Family status Intact *n* = 46 Separated *n* = 14	*N* = 61 Gender Female *n* = 54 Male *n* = 7 Age 12 to 18 years *M* = 14.1 Duration of illness 12.3 months Previous hospitalisations 25 days Using psychotropic medications *n* = 9 BMI percentile *M* = 7.2 Family status Intact *n* = 49 Separated *n* = 12	Expected Body Weight (EBW) Body Mass Index (BMI) The Eating Disorder Examination (EDE, v12; Cooper et al., 1989)	Multisite Randomised Control Trial (RCT)	Pre treatment End of treatment 12 months post treatment	For FBT, a weight gain of 2.65 kg by session three predicted full remission at end of treatment compared to a weight gain of 3.20 kg by session four for AFT. Despite this, early weight gain did not predict remission at the 12 month follow up for AFT or FBT. Weight gain was slightly more significant for participants in the FBT compared to the AFT treatment group. Furthermore, participants who demonstrate early weight gain were likely to drop out before treatment completion for AFT and FBT groups.	Does not explore participants who did not reach full remission (i.e., partial remission).
Le Grange, Lock et al. (2014) Relapse from remission at two- to four-year follow-up in two treatments for adolescent anorexia nervosa	DSM-IV excluding the amenorrhea criterion Anorexia Nervosa	*N* = 43 Gender NA Age 13 to 22 years *M* = 19.83 Using psychotropic medications *n* = 15	*N* = 36 Gender NA Age 13 to 22 years *M* = 19.54 Using psychotropic medications *n* = 16	Expected Body Weight (EBW) Body Mass Index (BMI) The Eating Disorder Examination (EDE, v12; Cooper et al., 1989) The Beck Depression Inventory (BDI; Beck et al., 1988) The Rosenberg Self-Esteem Scale (RSES; Rosenberg, 1965) Yale-Brown-Cornell Eating Disorder Scale (YBC-EDS; Mazure et al., 1994)	Multisite Randomised Control Trial (RCT)	Pre treatment End of treatment 6 months post treatment 12 months post treatment 24 months post treatment 36 months post treatment 48 months post treatments	Two participants; one from the AFT treatment group and one from the FBT treatment group relapsed after full-remission was achieved 12 months post treatment Ten new participants achieved remission (FBT *n* = 1; AFT *n* = 9) No differences were found between AFT and FBT in either relapse from full remission or new remission during 24 months and 36 month follow-ups.	The long-term study was not planned as part of the primary study; consequently, data was only available for 65.3% of the participants
Lock et al. (2010) Randomized clinical trial comparing family-based treatment with adolescent-focused individual therapy for adolescents with anorexia nervosa	DSM-IV excluding the amenorrhea criterion Anorexia Nervosa Subtype Binge-Eating/Purging *n* = 21 Restrictive *n* = 100	*N* = 60 Gender Female *n* = 56 Male *n* = 4 Age 12 to 18 years *M* = 14.7 Duration of illness 10.3 months Previous hospitalisations 29 days Using psychotropic medications *n* = 11 BMI percentile *M* = 5.2 Family status Intact *n* = 46 Separated *n* = 14	*N* = 61 Gender Female *n* = 54 Male *n* = 7 Age 12 to 18 years *M* = 14.1 Duration of illness 12.3 months Previous hospitalisations 25 days Using psychotropic medications *n* = 9 BMI percentile *M* = 7.2 Family status Intact *n* = 49 Separated *n* = 12	Expected Body Weight (EBW) Body Mass Index (BMI) The Eating Disorder Examination (EDE, v12; Cooper et al., 1989) The Beck Depression Inventory (BDI; Beck et al., 1988) The Rosenberg Self-Esteem Scale (RSES; Rosenberg, 1965) Schedule for Affective Disorder and schizophrenia for school-Age Children; K-SADS; (Kaufman et al., 1997)	Multisite Randomised Control Trial (RCT)	Pre treatment End of treatment 6 months post treatment 12 months post treatment	No differences were found for full remission (> 95% EBW) between AFT and FBT at the end of treatment. However, for the 6 month and 12 month follow ups FBT was significantly superior to AFT In addition, FBT was significantly superior to AFT for partial remission (>85% EBW) at end of treatment, yet this difference was indistinguishable at 6 month and 12 month follow ups	During the treatment administration participants were hospitalised in AFT (*n* = 32) and FBT (*n* = 9) EDE scores was higher in AFT group than FBT at baseline The participants in the FBT group were slightly younger than the participants in the AFT group
Robin, Siegel, Koepke et al., (1994) Family therapy versus individual therapy for adolescent females with anorexia nervosa	DSM-III-R Anorexia Nervosa	*N* = 11 Gender Female *n* = 11 Male *n* = 0 Age 12 to 19 years *M* = 13.9 Duration of illness <12 months BMI percentile *M* = 5.2 Weight *M* = 91.0 lbs	*N* =11 Gender Female *n* = 11 Male *n* = 0 Age 12 to 19 years *M* =14.7 Duration of illness <12 months BMI percentile *M* = 7.2 Weight *M* = 85.4 lbs	Expected Body Weight (EBW) Body Mass Index (BMI) The Beck Depression Inventory (BDI; Beck et al., 1988)	Randomised Control Trial (RCT)	Pre treatment 12 months post treatment	FBT was superior to AFT in participant weight gain and menstruation.	The study used weight gain as the outcome measure, rather than using the remission criteria from the DSM-III-R Weight gain is not always equivalent with recovery
Robin, Siegel, and Moye(1994) Family versus individual therapy for anorexia: impact on family conflict	DSM-III-R Anorexia Nervosa	*N* = 11 Gender Female *n* =11 Male *n* = 0 Age 12 to 19 years *M* = 13.9 Duration of illness <12 months Weight *M* = 86.8 pounds	*N* = 11 Gender Female *n* = 11 Male *n* = 0 Age 12 to 19 years *M* = 14.7 Duration of illness <12 months Weight *M* = 86.5 pounds	Expected Body Weight (EBW) Body Mass Index (BMI) The Beck Depression Inventory (BDI; Beck et al., 1988) Parent Adolescent Relationship Questionnaire, measuring family conflict (PARQ; Robin et al., 1990) Observed Family Conflict	Randomised Control Trial (RCT)	Pre treatment 12 months post treatment	FBT and AFT treatments reduced negative communication between family members and had a positive influence on parent-adolescent relationships Eating-related conflict significantly reduced and maintained at the 12 month follow-up	The study used weight gain as the outcome measure, rather than using the remission criteria from the DSM-III-R Weight gain is not always equivalent with recovery
Robin et al. (1999) A controlled comparison of family versus individual therapy for adolescents with anorexia nervosa	DSM-III-R Anorexia Nervosa	*N* = 18 Gender Female *n* = 18 Male *n* = 0 Age 11 to 20 years *M* = 13.4 Duration of illness <12 months BMI percentile *M* = 5.2 Weight *M*=86.8 pounds Family status Intact *n* = 46 Separated *n* = 14	*N* = 19 Gender Female *n* = 19 Male *n* = 0 Age 11 to 20 years *M* = 14.9 Duration of illness <12 months BMI percentile *M* = 7.2 Weight *M* = 86.5 pounds Family status Intact *n* = 49 Separated *n* = 12	Expected Body Weight (EBW) Body Mass Index (BMI) The Beck Depression Inventory (BDI; Beck et al., 1988) Eating Disorder Inventory (Garner et al., 1983) Eating Attitude Tests (EAT-26; Garner & Garfinkel, 1979) Parent Adolescent Relationship Questionnaire, measuring family conflict (PARQ; Robin et al., 1990)	Randomised Control Trial (RCT)	Pre treatment End of Treatment 12 months post treatment	FBT produced greater weight gain and higher rates of menstruation than AFT	The study used weight gain as the outcome measure, rather than using the remission criteria from the DSM-III-R. It is a limitation because weight gain is not always equivalent with recovery

### Quality Rating

The primary studies that explored weight increases or remission rates include Lock et al. (2010), Le Grange, Lock et al. (2014), and Robin, Siegel, and Moye (1994; 1999). However, most of the studies were secondary (i.e., using data from the primary studies). Each secondary study explored specific components identified in the three primary studies contributing to FBT’s and AFT’s effectiveness on adolescents and young adults diagnosed with AN reaching remission. Thus, the secondary studies add to the research and assist clinicians to identify the most effective treatment for AN. Furthermore, the three primary studies were deemed robust. All primary studies included utilised randomised control trials and sound psychometric assessments, and had a representative sample.

## Discussion

The present systematic review aimed to compare the efficacy of AFT and FBT in treating adolescents and young adults diagnosed with AN to reach and maintain full remission and inform psychotherapists working in the field of factors to consider in therapy delivery.

### Treatment Efficacy

Robin, Siegel, and Moye (1994) reported FBT and AFT were equally effective interventions in improving weight gain at the end of treatment. However, at the 12-month follow-up, the number of participants who met their target weight and began menstruating increased substantially for FBT and only mildly for AFT. Robin et al. (1999) results support Robin, Siegel, and Moye (1994) outcomes, indicating pronounced differences between FBT and AFT at the end of treatment, yet, FBT demonstrated meaningful improvements over AFT at the 12-month follow-up.

In comparison, Lock et al. (2010) indicated that FBT was superior to AFT at end of treatment, with significantly more participants in FBT reaching partial and full remission. Lock et al. (2010) also found at the 6-month and 12-month follow-ups, FBT remained superior to AFT for full remission; however, they were equivalent for partial remission rates. Remission rates were significantly lower than Robin, Siegel, and Moye (1994, 1999) studies. Despite the low remission rates, Lock et al. (2010) used definitive cut-off markers from the DSM-IV diagnostic criteria, as opposed to relying on BMI alone (i.e., full revision >95% EBW and partial remission >85% EBW). The differences may also result from variance among participant sizes. Lock et al. (2010) had a large sample (*N* = 121) and a moderate main effect when compared to the low power described in Robin, Siegel, and Moye (1994; *N* = 22) and Robin et al. (1999; *N* = 37).

Le Grange, Lock et al. (2014) tested the efficacy of FBT and AFT up to four years after Lock et al. (2010) treatments. Le Grange, Lock et al. (2014) found at the 24-month follow-up, more participants in the AFT than FBT relapsed after full remission, yet a significantly larger proportion of new participants in AFT had achieved full remission. Furthermore, relapse from full remission and new participants meeting full remission was not distinguishable between FBT and AFT treatments at the 36-month and 48-month follow-up (Le Grange, Lock et al., 2014). Le Grange, Lock et al. (2014) also explored early weight gain factors likely to predict remission at the end of treatment. They found that participants who gained a minimum of 2.65 kg by week three for FBT and 3.20 kg by week four for AFT were likely to reach partial or full remission at the end of treatment. Results highlight that early weight gain was not a predictor of partial or full remission for AFT or FBT; however, this was the only study that explored remission rates beyond the 12-month follow-up period.

Similarly, Le Grange et al., 2012 was the only study that tested the difference between baseline scores and their relationship in predicting remission. Participants with higher baseline scores benefited more from FBT than AFT, expressed by higher partial and full remission rates. Also, they compared AN subtypes and found that Binge-Eating/Purging AN responded significantly less to either treatment than Restrictive AN.

Across the primary studies, there were differences in treatment retention rates. In Robin, Siegel, and Moye’s (1994), retention rates were 100 and 82% for FBT and AFT, respectively; Lock et al. (2010) retention rates were 84% for FBT and 92% for AFT; Robin et al. (1999) retained 100% of for FBT and AFT. Regarding Robin, Siegel, Koepke et al. (1994), differences may be due to FBT requiring more treatment hours than AFT. Also, weight gain was slightly superior for participants in FBT than AFT, potentially demonstrating the difference. Alternatively, Lock et al. (2010) and Le Grange, Accurso et al. (2014) noted participants demonstrating early weight gain were likely to drop out before treatment completion; however, no patterns or differences were evident in drop-out rates between the treatments.

Furthermore, participants requiring hospitalisation during the study stopped treatment and continued when discharged. Lock et al. (2010) found more participants were hospitalised from AFT compared to FBT, with a median number of 12 and 10 days for FBT and AFT, respectively. Weight gain during hospitalisation was a median of 1.0 kg for FBT and 1.7 kg for AFT participants. In comparison, Robin et al. (1999) had higher hospital admissions rates for FBT than for AFT. Similarly, Robin, Siegel, and Moye (1994) indicated more FBT participants than AFT were hospitalised, with an average of 26.4 days. The mean days spent in hospital for Robin et al. (1999) study is unknown; however, participants were discharged when they reached 80% of their target weight; this likely inflated the results. Furthermore, most of Lock et al.'s (2010) hospitalisations occurred in the first four weeks of treatment. Le Grange, Lock et al. (2014) found that weight increases during this period predicted remission at the end of treatment, demonstrating a limitation.

### External Influences on Remission Rates

Each study investigated external factors that could influence the effectiveness of FBT and AFT. Accurso et al. (2014) measured psychological symptoms of AN regarding dietary restraint, depression, and perceptions of self-esteem, body image, and weight. They found depressive and dietary symptoms significantly reduced regardless of treatment. A similar effect was found in Robin et al.’s (1999) study. Unhealthy perceptions of daily food intake notably reduced for FBT and AFT; however, at the 12-month follow-up, AFT was superior for reducing unrealistic perceptions. Nevertheless, depression scores were much higher for FBT than AFT, contrasting with Accurso et al.’s (2014) findings. Differences between the two studies may be due to differences in each treatment’s delivery protocol. For example, in FBT, the client is not considered competent enough to control their daily food intake. Instead, the parents/caregivers take on the responsibility, which may influence the client’s perceptions of self-efficacy and mental well-being (Byrne et al., 2015). Byrne et al. (2015) explored differences in adolescent and parental self-efficacy ratings at full remission. Families receiving FBT indicated notable increases in parental self-efficacy, forecasting subsequent weight gain. However, participants’ self-efficacy ratings did not predict their weight gain.

Ciao et al., 2015 measured levels of family conflict and subsequent effects on remission using data from Lock et al. (2010) primary study. Ciao et al., 2015 found developments in communication and support were associated with full remission at end of treatment, irrespective of initial level of family functioning in either treatment. In comparison, in Robin et al. (1994), AFT and FBT produced notable reductions in negative communication. Specifically, FBT demonstrated remarkably higher interactions and improvements in eating-related conflict, which were maintained at the 12-month follow-up. Although AFT’s primary focus is not on family support and connection, significant communication improvements were associated with weight increase (Robin et al., 1994).

Forsberg (2011) and Forsberg et al. (2014) compared the level of therapeutic rapport and its effect on remission. Consistently, high levels of therapeutic rapport were found across treatment groups during each period; however, the AFT group had significantly stronger alliance scores at end of treatment. Additionally, a strong therapeutic alliance was identified to predict partial remission by end of treatment, yet it did not predict full remission. Forsberg (2011) and Forsberg et al. (2014) included only 78 out of the 121 participants from Lock et al. (2010) primary study due to audio quality difficulties, highlighting limitations as they did not have adequate power to address their research question.

## Conclusions

Across the three primary studies, FBT resulted in significant weight gain and higher partial and full remission rates than AFT, demonstrating its superiority in treating AN in adolescents and young adult samples, in one instance at least up to 4 years. Hence, FBT in the current sample of empirical literature demonstrates greater long-term efficaciousness in the remission of AN. However, AFT may be a suitable treatment when FBT is not feasible (e.g., there is a high family disconnect).

Despite FBT and AFT delivery, a significant proportion of participants in the three primary studies did not achieve their target weight or full remission, indicating that both treatments may not be effective in all circumstances. Additionally, the therapeutic relationship, anxiety and depression symptoms, hospitalisation, perceptions of family conflict, self-efficacy, body image, and weight influence participants’ remission across FBT and AFT. Psychology practitioners are recommended to consider including an additional therapeutic technique also known to positively influence Anorexia Nervosa weight gain and remission (e.g., Cognitive-Behaviour Therapy; Nyman-Carlsson et al., 2020).

Furthermore, there are only three primary studies, and the most recently published article is from 2010; thus, limitations arise when extrapolating data from such a small number of articles during a similar period. Such limitations are concerning, as additional factors may impact treatment nowadays (e.g., social media use and social change). Also, all primary studies were conducted in the United States of America, restricting the generalisability of treatment effects to other populations as cross-cultural considerations are not regarded. It is recommended that other research groups conduct longitudinal studies, exploring AFT and FBT’s effectiveness in attaining and maintaining remission among adolescents and young adults diagnosed with AN. Comparing the effectiveness of AFT to the current gold standard approach (FBT) may provide clients and families with a strong treatment alternative and improve remission rates by acknowledging that not all clients respond positively to all treatment approaches. Such information would aid in informing treating practitioners of AFT and FBT’s potential and limitations in reducing the prevalence and mortality associated with AN.

## Data Availability

Data sharing not applicable to this article as no datasets were generated or analyzed during the current study.

## Appendix

### List of Abbreviations

AFT: Adolescent-Focused Therapy
AN: Anorexia Nervosa
BDI: Beck Depression Inventory
BN: Bulimia Nervosa
BMI: Body Mass Index
BSQ: Body Shape Questionnaire
DSM-III-R: Diagnostic and Statistical Manual of Mental Disorders, Third Edition, Revised
DSM-IV: Diagnostic and Statistical Manual of Mental Disorders, Fourth Edition
EAT-26: Eating Attitude Test, version 26
EBW: Expected Body Weight
EDE: Eating Disorder Examination
FAD: Family Assessment Device
FBT: Family-Based Therapy
GES: General Self-Efficacy Scale
K-SADS: Schedule for Affective Disorders and Schizophrenia for School-Age Children
PARQ: Parent-Adolescent Relationship Questionnaire
PRISMA: Preferred Reporting Items for Systematic Reviews and Meta-Analyses
RSES: Rosenberg Self-Esteem Scale
WSAS: Work and Social Adjustment Scale
WAI: Working Alliance Inventory
YBC-EDS: Yale-Brown-Cornell Eating Disorder Scale

## References

[bibr1-00332941241226687] Accurso E. C. Ciao A. C. Fitzsimmons-Craft E. E. Lock J. D. Le Grange D. (2014). Is weight gain really a catalyst for broader recovery? The impact of weight gain on psychological symptoms in the treatment of adolescent anorexia nervosa. Behaviour Research and Therapy, 56, 1–6. 10.1016/j.brat.2014.02.006 24632109 PMC4019781

[bibr2-00332941241226687] American Psychiatric Association . (1987). Diagnostic and statistical manual of mental disorders, Dsm-Iii-R (Revised ed.). Amer Psychiatric Pub Inc. 10.1176/appi.books.9780890420188.dsm-iii-r

[bibr3-00332941241226687] American Psychiatric Association . (1994). Diagnostic and Statistical Manual of Mental Disorders, 4th Edition, (DSM-IV) (4th ed.). American Psychiatric Association. 10.1176/appi.books.9780890420249.dsm-iv-tr

[bibr4-00332941241226687] American Psychiatric Association . (2013). Desk reference to the diagnostic criteria from DSM-5. Independently published. 10.1176/appi.books

[bibr5-00332941241226687] Arcelus J. Mitchell A. J. Wales J. Nielsen S. (2011). Mortality Rates in Patients with Anorexia Nervosa and Other Eating Disorders. Archives of General Psychiatry, 68(7), 724. 10.1001/archgenpsychiatry.2011.74 21727255

[bibr43-00332941241226687] Beck A Steer R Garbin M (1988) (In press). Psychometric Properties of the Beck Depression Inventory: 25 Years of Evaluation. Clinical Psychology Review, 8, 77–100. 10.1016/0272-7358(88)90050-5

[bibr6-00332941241226687] Byrne C. E. Accurso E. C. Arnow K. D. Lock J. le Grange D. (2015). An exploratory examination of patient and parental self-efficacy as predictors of weight gain in adolescents with anorexia nervosa. International Journal of Eating Disorders, 48(7), 883–888. 10.1002/eat.22376 25808269 PMC4845658

[bibr7-00332941241226687] Chen E. Y. Weissman J. A. Zeffiro T. A. Yiu A. Eneva K. T. Arlt J. M. Swantek M. J. (2016). Family-based therapy for young adults with Anorexia Nervosa restores weight. International Journal of Eating Disorders, 49(7), 701–707. 10.1002/eat.22513 27037965 PMC7350506

[bibr9-00332941241226687] Cooper P. J. Taylor M. J. Cooper Z. Fairbum C. G. (1987). The development and validation of the body shape questionnaire. International Journal of Eating Disorders, 6(4), 485–494. 10.1002/1098-108x

[bibr8-00332941241226687] Ciao A. C. Accurso E. C. Fitzsimmons-Craft E. E. Lock J. le Grange D. (2015). Family functioning in two treatments for adolescent anorexia nervosa. International Journal of Eating Disorders, 48(1), 81–90. 10.1002/eat.22314 24902822 PMC4382801

[bibr10-00332941241226687] Cooper Z. Cooper P. J. Fairburn C. G. (1989). The validity of the eating disorder examination and its subscales. British Journal of Psychiatry, 154(6), 807–812. 10.1192/bjp.154.6.807 2597887

[bibr11-00332941241226687] Epstein N. B. Baldwin L. M. Bishop D. S. (1983). The McMaster family assessment device. Journal of Marital and Family Therapy, 9(2), 171–180. 10.1111/j.1752-0606.1983.tb01497.x

[bibr13-00332941241226687] Forsberg S. (2011). The relationship between therapeutic alliance and treatment outcome in a comparative study of individual and family therapy for adolescent anorexia nervosa (Order No. 3591823). Available from ProQuest Dissertations & Theses Global. (1433375288). https://ezproxy.bond.edu.au/login?url=https://www.proquest.com/dissertations-theses/relationship-between-therapeutic-alliance/docview/1433375288/se-2?accountid=26503

[bibr14-00332941241226687] Forsberg S. LoTempio E. Bryson S. Fitzpatrick K. K. Le Grange D. Lock J. (2014). Parent-therapist alliance in family-based treatment for adolescents with anorexia nervosa. European Eating Disorders Review, 22(1), 53–58. 10.1002/erv.2242 23861093

[bibr15-00332941241226687] Garner D. M. Garfinkel P. E. (1979). The eating attitudes test: An index of the symptoms of anorexia nervosa. Psychological Medicine, 9(2), 273–279. 10.1017/s0033291700030762 472072

[bibr16-00332941241226687] Garner D. M. Garfinkel P. E. (1984). Handbook of psychotherapy for anorexia nervosa and bulimia (2nd Printing ed.). The Guilford Press.

[bibr17-00332941241226687] Garner D. M. Olmstead M. P. Polivy J. (1983). Development and validation of a multidimensional eating disorder inventory for anorexia nervosa and bulimia. International Journal of Eating Disorders, 2(2), 15–34. 10.1002/1098-108x(198321)2:2<15::aid-eat2260020203>3.0.co;2-6

[bibr44-00332941241226687] Kaufman J Birmaher B Brent D Rao U Flynn C Moreci P Williamson D Ryan N (1997) (In press). Schedule for Affective Disorders and Schizophrenia for School-Age Children-Present and Lifetime Version (K-SADS-PL): initial reliability and validity data. J Am Acad Child Adolesc Psychiatry, 36(7), 980–988. https://10.1097/00004583-199707000-00021 9204677 10.1097/00004583-199707000-00021

[bibr18-00332941241226687] Keski-Rahkonen A. Raevuori A. Hoek H. W. (2008). Epidemiology of eating disorders: An update (Annual Review of Eating Disorders: 1 ed.). CRC Press. https://www.taylorfrancis.com/chapters/edit/10.4324/9781315380063-11/epidemiology-eating-disorders-update-anna-keski-rahkonen-anu-raevuori-hans-hoek

[bibr19-00332941241226687] Le Grange D. Accurso E. C. Lock J. Agras S. Bryson S. W. (2014). Early weight gain predicts outcome in two treatments for adolescent anorexia nervosa. International Journal of Eating Disorders, 47(2), 124–129. 10.1002/eat.22221 24190844 PMC4341963

[bibr20-00332941241226687] Le Grange D. Lock J. Accurso E. C. Agras W. S. Darcy A. Forsberg S. Bryson S. W. (2014). Relapse from remission at two- to four-year follow-up in two treatments for adolescent anorexia nervosa. Journal of the American Academy of Child & Adolescent Psychiatry, 53(11), 1162–1167. 10.1016/j.jaac.2014.07.014 25440306 PMC4254507

[bibr21-00332941241226687] Le Grange D. Lock J. Agras W. S. Moye A. Bryson S. W. Jo B. Kraemer H. C. (2012). Moderators and mediators of remission in family-based treatment and adolescent focused therapy for anorexia nervosa. Behaviour Research and Therapy, 50(2), 85–92. 10.1016/j.brat.2011.11.003 22172564 PMC3260378

[bibr22-00332941241226687] Lock J. Le Grange D. Agras W. S. Moye A. Bryson S. W. Jo B. (2010). Randomized clinical trial comparing family-based treatment with adolescent-focused individual therapy for adolescents with anorexia nervosa. Archives of General Psychiatry, 67(10), 1025. 10.1001/archgenpsychiatry.2010.128 20921118 PMC3038846

[bibr23-00332941241226687] Mazure C. M. Halmi K. A. Sunday S. R. Romano S. J. Einhorn A. M. (1994). The Yale-Brown-Cornell eating disorder scale: Development, use, reliability and validity. Journal of Psychiatric Research, 28(5), 425–445. 10.1016/0022-3956(94)90002-7 7897615

[bibr24-00332941241226687] McGorry P. (2020). The economic and social impact of eating disorders in Australia. (Paying The Price ed.). The Butterfly Foundation. https://butterfly.org.au/wp-content/uploads/2020/06/Butterfly_Report_Paying-the-Price.pdf

[bibr26-00332941241226687] Moher D. Liberati A. Tetzlaff J. Altman D. G. PRISMA Group . (2009). Preferred reporting items for systematic reviews and meta-analyses: The PRISMA statement. Annals of Internal Medicine, 151(4), 264. 10.7326/0003-4819-151-4-200908180-00135 19622511

[bibr27-00332941241226687] Mundt J. C. Marks I. M. Shear M. K. Greist J. M. (2002). The Work and social adjustment scale: A simple measure of impairment in functioning. British Journal of Psychiatry, 180(5), 461–464. 10.1192/bjp.180.5.461 11983645

[bibr28-00332941241226687] Nyman-Carlsson E. Norring C. Engström I. Gustafsson S. A. Lindberg K. Paulson-Karlsson G. Nevonen L. (2020). Individual cognitive behavioral therapy and combined family/individual therapy for young adults with anorexia nervosa: A randomized controlled trial. Psychotherapy Research, 30(8), 1011–1025. 10.1080/10503307.2019.1686190. 31709920

[bibr29-00332941241226687] Robin A. L. Foster S. L. (1989). Parent-adolescent conflict: A behavioral family Systems approach. Guilford Press.

[bibr30-00332941241226687] Robin A. L. Koepke T. Moye A. (1990). Multidimensional assessment of parent-adolescent relations. Psychological Assessment: A Journal of Consulting and Clinical Psychology, 2(4), 451–459. 10.1037/1040-3590.2.4.451

[bibr31-00332941241226687] Robin A. L. Siegel P. T. Koepke T. Moya A. W. Tice S. (1994). Family therapy versus individual therapy for adolescent females with anorexia nervosa. Journal of Developmental & Behavioral Pediatrics, 15(2), 111–116. 8034762. 8034762

[bibr45-00332941241226687] Robin A Siegel P Koepke T Moye A Tice S (1994) (In press). Family Therapy Versus Individual Therapy for Adolescent Females with Anorexia Nervosa. J Dev Behav Pediatr, 15(2), 111–116. 8034762. 8034762

[bibr32-00332941241226687] Robin A. L. Siegel P. T. Moye A. (1994). Family versus individual therapy for anorexia: Impact on family conflict. International Journal of Eating Disorders, 17(4), 313–322. 10.1002/1098-108x 7620470

[bibr33-00332941241226687] Robin A. L. Siegel P. T. Moye A. W. Gilroy M. Dennis A. B. Sikand A. (1999). A controlled comparison of family versus individual therapy for adolescents with anorexia nervosa. Journal of the American Academy of Child & Adolescent Psychiatry, 38(12), 1482–1489. 10.1097/00004583-199912000-00008 10596247

[bibr34-00332941241226687] Rosenberg M. (1965). Rosenberg self-esteem scale (RSES) [Database record]. APA PsycTests.

[bibr35-00332941241226687] Schantz P. M. Tsang V. C. (2003). The US centers for Disease Control and Prevention (CDC) and research and control of cysticercosis. Acta Tropica, 87(1), 161–163. 10.1016/s0001-706x(03)00039-1 12781391

[bibr36-00332941241226687] Sukmak V. Sirisoonthon A. Meena P. (2001). The validity of the general perceived self-efficacy scale. Journal of the Psychiatric Association of Thailand, 47(1), 31–37. https://psychiatry.or.th/JOURNAL/vol471/47-1-05.pdf

[bibr37-00332941241226687] Tichenor V. Hill C. E. (1989). A comparison of six measures of working alliance. Psychotherapy: Theory, Research, Practice, Training, 26(2), 195–199. 10.1037/h0085419

